# X-Chromosome Inactivation and Related Diseases

**DOI:** 10.1155/2022/1391807

**Published:** 2022-03-27

**Authors:** Zhuo Sun, Jinbo Fan, Yang Wang

**Affiliations:** ^1^Xi'an Key Laboratory of Pathogenic Microorganism and Tumor Immunity, Xi'an Medical University, No. 1 XinWang Road, Weiyang District, Xi'an 710021, Shaanxi, China; ^2^Institute of Basic Medical Sciences, Xi'an Medical University, No. 1 XinWang Road, Weiyang District, Xi'an 710021, Shaanxi, China

## Abstract

X-chromosome inactivation (XCI) is the form of dosage compensation in mammalian female cells to balance X-linked gene expression levels of the two sexes. Many diseases are related to XCI due to inactivation escape and skewing, and the symptoms and severity of these diseases also largely depend on the status of XCI. They can be divided into 3 types: X-linked diseases, diseases that are affected by XCI escape, and X-chromosome aneuploidy. Here, we review representative diseases in terms of their definition, symptoms, and XCI's role in the pathogenesis of these diseases.

## 1. X-Chromosome Inactivation, Escape, and Skewing

During XCI, many epigenetic events take place and ensure the compacted heterochromatin structure of the inactive X chromosome (X inactive, Xi) to silence most genes on the chromosome. XIST lncRNA is expressed and coats the Xi in cis [1, 2], which starts a cascade of events including substitution of macroH2A [3], the removal of active histone modifications [4, 5], the addition of repressive histone modifications [6–9], CpG islands methylation [10], and heterochromatin protein recruitment [11, 12]. As a result, the Xi is compacted into a rounder shape compared to the more flat structure of the active X chromosome (X active, Xa) [13].

Not all genes on the Xi are in a repressed state. It is estimated that about 15%–30% of all the genes on the Xi escape gene repression and are expressed [14, 15]. For different individuals, ages, and cell types, the gene escape patterns are diverse [14, 16]. Some genes are found to be escaping in most cell types, others are highly variable depending on the origin of cells. The occurrence of escaped genes has been found to be essential in the pathogenesis of many diseases including autoimmune diseases [17] and cancer [18–21].

Skewed X-chromosome inactivation or X-chromosome inactivation skewing describes the phenomenon when more than 75% of cells in an individual chose the X chromosome from one parent as the Xi. This occurs since the choice of which X chromosome to silence is random and it takes place early in the gastrulation stage, so when the choice is at the tail end of the normal distribution, or if alleles of specific genes from one parent's origin render the cells more robust, it may lead to skewing of the X-chromosome inactivation, instead of the 50% completely random choice [22, 23]. It is estimated that 1.5%–23% of females have skewed X inactivation [24–26]. The direction and degree of XCI skewing may influence the severity of some diseases including haemophilia B [27, 28], dyskeratosis congenita [29], Duchenne muscular dystrophy [30], myotubular myopathy [31], and Fabry disease (FD) [32, 33], which will be discussed more in detail in this review.

Many diseases have been found to be related to the XCI process. They can be roughly categorized to 3 types: (1) X-linked gene diseases, whose severity is greatly influenced by the direction and degree of X-inactivation skewing; here, we review FD that can be categorized to this type; (2) diseases that have higher occurrence in female population due to the presence of an extra pair of X chromosome and the possibilities to have escaped gene expression from the Xi: systemic lupus erythematosus (SLE) will be reviewed in this paper as an example; (3) X-chromosome aneuploidy: Turner syndrome (TS, 45, X), triple X syndrome (47, XXX), and Klinefelter syndrome (47, XXY) belong to this type. These diseases will be reviewed in terms of their clinical symptoms, pathogenic mechanism, and the role of XCI in the clinical presentation.

## 2. XCI-Related Diseases

### 2.1. X-Linked Diseases

For almost all X-linked diseases, clinical manifestation is more severe in males than in females. Female carriers are either asymptomatic or show milder phenotypes compared to males. Sex differences in these X-linked diseases are due to XCI [34]. Males are hemizygous for most X-linked genes; thus, a male carrier of a mutant allele is usually affected with full presentation of the disease clinical features. Females have two types of cells in terms of their XCI status: those whose maternal X is active and those whose paternal X is active. Therefore, even if a female carries one copy of mutant allele, she probably still does not have full clinical presentation because a portion of the female cells has the mutant X inactivated. Data from OMIM show that there are more than 500 X-linked diseases that affect males more severely. X-linked retinitis pigmentosa [35, 36], Duchenne muscular dystrophy [37], FD, and fragile X syndrome all belong to this category.

Among those X-linked diseases with higher male susceptibility, some rarely affect females, whereas others can present severe symptoms in female heterozygotes. There are mainly two factors behind whether female heterozygotes will have clinical manifestation: protein transfer and cell selection [38]. Protein transfer is the process when variant cells could not make functional proteins but wildtype cells can transfer functional proteins into those deficient cells to make up for the loss. Cell selection is the process when there is a growth advantage for either the wildtype cells or mutant cells because of the mutant phenotype, the other cell population dies off gradually, making disease manifestation not evident or very severe, respectively.

Hunter syndrome and FD are both X-linked lysosomal enzyme diseases. Hunter syndrome rarely affects females [39], whereas FD can present severe symptoms in female patients. The difference in female susceptibility lies in the different ability for cells to share the lysosomal enzyme [40]. The enzyme iduronic sulfatase loss in Hunter syndrome can be readily supplied by nearby wildtype cells, whereas the mature form of *ɑ*-galactosidase A (*ɑ*-GAL A) is hard to uptake for mutant cells in FD [41].

There are X-linked diseases that only or mainly affect females. Those disease variants usually cause loss of an essential protein completely, so that males are lethal *in utero*, leaving females to be the major sex to be afflicted with these diseases. Cornelia de Lange 2 (with SMC1A truncating variants) [42] and CHILD syndrome [43] only affect females since male carriers are lethal *in utero*. Other diseases affect some males due to a milder form of mutant or mosaic. Rett syndrome [44], incontinentia pigmenti type 2 [45], and focal dermal hypoplasia [46] all belong to this type.

In the next part, FD will be discussed in detail as an example to show how XCI is involved in the pathogenesis of X-linked diseases. FD is caused by mutations in the GLA gene which codes for the *ɑ*-GAL A enzyme. *ɑ*-GAL A breaks down globotriaosylceramide and glycosphingolipids in the lysosome for recycling in cell metabolism, and decreased activity or loss of *ɑ*-GAL A leads to buildup of those molecules in the lysosome which can cause multisystemic effects in patients [47]. *ɑ*-GAL A is abundant in the kidney and vascular tissues, and key manifestation of FD includes malfunction of the kidney and heart. The estimated incidence of FD is 1 in 117,000 [48].

The disease phenotype depends on residual enzyme activity: less than 1% of normal activity results in classic FD, and levels between 1% and 30% leads to atypical forms of FD (also called late-onset FD). Classic FD mainly affects males. Classic FD patients usually present symptoms early in childhood that include acute pain in extremities and fatigue, hypohidrosis [49], neuropathic pain in the hands and feet, angiokeratomas in the lower abdomen and bathing trunk area [50], gastrointestinal problems, and cornea verticillata [51, 52]. Because of the residual *ɑ*-GAL A activity, atypical FD patients develop symptoms much later in life. Some develop multiple symptoms as young adults, while others only show signs of FD in specific organs such as the heart and kidneys. Heterozygous females have 0–100% of normal plasma *ɑ*-GAL A activity and can have symptoms that range from mild to severe depending on their skewing of XCI [53].

Males are usually severely affected, whereas clinical presentation in female patients is more variable [53, 54]. Females usually develop symptoms in their adulthood which is much later than males, and symptoms are usually milder [55]. This can lead to misdiagnosis in female patients. Since male has one copy of X chromosome, defect in the GLA gene can cause FD in males, whereas female has two X chromosomes and depending on X chromosome being inactivated and skewing of XCI, female mutant gene carriers can have a spectrum of clinical presentation from completely nonsymptomatic to severe symptoms as seen in males [56].

In female FD patients, both random XCI and skewed XCI are observed. In a study that evaluated XCI pattern of four different tissues from female FD patients, random XCI is observed in 71% of samples and skewed XCI in 29% of samples [56]. Other studies have found similar ratios [33, 57]. For patients with random XCI, disease presentation usually worsens severely with age, which is partially due to inefficient protein transfer between wildtype and affected cells. For patients with skewed XCI, predominant expression of the mutant GLA allele usually results in early onset and rapid progression in FD, whereas the favored expression of the wildtype GLA allele is associated with mild phenotype and little progression over time.

For male patients, diagnosis can be confirmed by low *ɑ*-GAL A activity in leukocytes, whereas for female patients, *ɑ*-GAL A activity can range from very low to normal levels. Therefore, gene sequencing is the gold standard for diagnosis in females [58]. It has been shown that level of *ɑ*-GAL A cannot predict the severity of symptoms well in female patients, and some researchers argue that the enzyme deficiency may result in other pathogenetic mechanisms [59–61].

It was believed that skewed XCI is the main reason for phenotype variability in female heterozygotes [32, 33, 56, 62], but recently some studies have come at the conclusions that skewed XCI could not explain all cases of severe FD and is not the main factor in the variable clinical presentation of FD in females [57, 63, 64].

One factor that might have complicated the results is the tissues chosen for analyzing the XCI skewing. Most studies chose easily accessible leukocytes, urinary cells, and buccal epithelia, rather than the tissues from affected organs, such as cardiac and renal tissues. Also, XCI skewing pattern could be different in different tissues from the same subject [65]. Examining samples from affected tissues would give the best perspective on XCI skewing's role in phenotype variability in female patients, but would require invasive biopsies.

Since some female patients with severe symptoms have random XCI, it points to factors other than XCI skewing that contribute to FD disease severity in heterozygous females. The nature of mutation could be essential in determining disease severity. Mutations that result in complete loss of the functional protein result in severe phenotypes, whereas missense mutations could result in mild phenotype or late-onset presentation. Even for family members with the same mutation, there could be vastly different clinical expression [61, 66]. It is likely that besides XCI skewing and nature of mutation, the variability in female patients is dependent on other genetic, epigenetic, and environment factors as well [58].

### 2.2. Diseases That Are Affected by XCI Escape or X-Chromosome Dosage Effect

Another type of diseases that are affected by XCI are due to X-inactivation escape. The escape and thus overexpression of some genes in female cells could be related to disease presentation and sex bias in those diseases. These include autoimmune diseases (SLE and autoimmune thyroid diseases [67, 68]) and some psychiatric disorders (bipolar disorder and major depression [69]).

Here, SLE will be discussed in detail as an example to show how XCI is involved in the pathogenesis of diseases affected by XCI escape and X-chromosome dosage effect. Many autoimmune diseases have sex bias where number of female patients is significantly higher than that of male patients. Many factors contribute to the sex bias including difference in innate immunity, immune response intensity [70], and hormones [71]. Many genetic risk loci have been discovered to be associated with SLE predisposition, some of which are X-linked. The extra pair of X chromosome, the possibility for these genes to escape, and a higher amount of the gene product in females than males contribute to sex bias of this disease.

SLE is a chronic autoimmune disease which leads to variable clinical presentations depending on the major organ affected. The word erythematosus refers to the rash that patients usually have on their skin. SLE incidence ranges from 2.2 to 23.1/100,000 person-years globally [72, 73], with the highest estimated incidence in North America. Women have higher prevalence of SLE [74, 75], and people of African ethnicity have higher incidence and prevalence than Caucasians [76].

For people with predisposition for SLE, antinuclear antibodies are produced and form immune complexes with nuclear antigen, which then deposit in tissues and cause inflammation. Antibodies against red blood cells and white blood cells could also be produced and result in type II hypersensitivity. There are more than 80 genetic predispositions that have been discovered to date, such as TREX1 [77], C8orf13-BLK [78], ITGAM-ITGAX [78], IL10 [79], TNIP1 [79], and IKZF1 [80] (the abovementioned gene loci are not X-linked). For most patients, SLE is caused by mutations in several genes rather than a single locus.

Besides genetic factors, there are also epigenetic factors that are essential in the disease pathogenesis. There is around 70%–75% discordance of SLE incidence between identical twins [81, 82]; this could be due to the different epigenetic landscape for these twins and also different X-inactivation pattern [83]. X-linked genes that are related to onset of SLE might be differentially inactivated among different cells, tissues, and individuals, contributing to the different onset of SLE in identical twins [84].

Symptoms of SLE include fatigue, fever, painful joints, rashes (especially butterfly-shaped rash on the cheek), and sensitivity to sun [85]. Since there are a variety of general and specific symptoms, diagnosis of SLE is difficult. Patients are diagnosed by adding scores from 10 clinical domains: constitutional, cutaneous, arthritis, neurological, serositis, haematological, renal, antiphospholipid antibodies, complement proteins, and highly specific antibodies [86]. SLE is characterized by periods of flare-ups and remittance, and treatments mainly involve immunomodulation and immunosuppression drugs and are targeted at preventing and limiting the severity of flare-ups.

Ratio of women with SLE to men is estimated to be 9 : 1 to 11 : 1. There could be several factors that contribute to the gender difference in disease susceptibility. These factors are hormones [87, 88], X-chromosome dosage factor, and X-linked gene overexpression.

It is shown that sex steroids can regulate autoimmune regulator (AIRE) locus expression, which in turn affects susceptibility to autoimmunity diseases [89, 90]. The fact that there is big increase of SLE incidence and prevalence in postpubertal females than males and prepubertal females also implies that sex hormones play an important role in the onset of SLE [91, 92].

The hypothesis that X-chromosome dosage is a contributing factor in SLE sex bias comes from the observations of SLE incidence in X-chromosome aneuploidies. Women with TS (45, X) are underrepresented compared to karyotypically normal women (46, XX) in SLE [93]. The risk of SLE in Klinefelter syndrome (47, XXY) males is 14-fold higher than karyotypically normal men (46, XY) [94]. The estimated prevalence of SLE in women with (47, XXX) is 2.5 times higher than women with normal karyotype and is 25 times higher than men [95], suggesting that dosage of X chromosome could be related to disease pathogenesis. Evidence from mouse models also indicates that an extra X chromosome is an important contributing factor in female bias in autoimmunity [96, 97]. It is important to note that chromosome dosage factor could be in part due to X-linked gene escape [98], which is the factor that is discussed next.

Overexpression of several X-linked genes has been shown to be involved in SLE pathogenesis, such as CD40LG [99], CXCR3 [100], KDM6A [101], CXorf21 [98], MECP2 [102, 103], IRAK1 [75, 103], TLR7(Toll-like receptor 7) [104, 105], GPR173 [106], and PRPS2. Several of them are immunity genes, including CD40LG, CXCR3, CXorf21, IRAK1, and TLR7. There could be 3 different scenarios of these X-linked gene overexpression, whether transcription from the active X allele is enhanced or there is escape from the inactive X allele or both. For the scenarios where XCI escape is involved in overexpression, it contributes to sex bias. Females by having the extra X chromosome have chances of XCI escape, whereas males do not.

Several studies have looked at the overexpression origin. TLR7 binds RNA in endosomes and activates the interferon response. TLR7 overexpression is observed in male patients, which is due to a SNP that increased transcription of the gene on the active X chromosome [104]. Another study found that there is biallelic expression of TLR7 in both female normal and SLE patient B cell lines, which means TLR7 is an escape even in healthy individuals [17]. The abovementioned studies show that TLR7 overexpression can come from both enhancing Xa allele expression and escape from Xi. CXorf21 is another immunity-related gene that shows XCI escape and has female-biased expression [98]. These are two examples of X-linked immunity gene escape contributing to sex bias in SLE.

A few other of these X-linked genes show overexpression only in female SLE patients but not in males, such as CD40LG [107, 108] and CXCR3 [108], which means the overexpression comes from the Xi, rather than Xa. Demethylation of the promoter region is also observed, which suggests that overexpression originates from XCI escape.

It has recently been observed that inactive X chromosome in SLE patient B cells have dramatic reduction in heterochromatic modifications, predisposing X-linked immunity gene escape [109]. This has provided further mechanistic insight as to how X-linked genes might contribute to SLE pathogenesis and sex bias.

### 2.3. X-Chromosome Aneuploidy

X-chromosome aneuploidy results in disease phenotypes in human: TS (45, X), Klinefelter syndrome (47, XXY), and triple X syndrome (47, XXX). For both Klinefelter syndrome and triple X syndrome, only one X chromosome remains active and all extra pairs of X chromosomes are inactivated. It is hypothesized that overexpression of escape genes results in the phenotypic abnormalities seen in those diseases [110]. The expression would be lower in TS due to haploinsufficiency. Indeed, SHOX gene, which escapes XCI, has been associated with tall stature in Klinefelter syndrome and triple X syndrome and short stature in TS [111, 112]. On the other hand, increased expression is also observed for some X-linked genes with decreasing X-chromosome dosage [113], indicating a compensatory mechanism in the complex relationship between X-chromosome dosage and X-linked gene expression level.

There is complex and diverse comorbidity associated with X-chromosome aneuploidy diseases, and identifying causal genes for different phenotypes has been difficult [114]. However, recent studies of genome-wide DNA methylation profile and transcriptome in patients revealed that there is DNA hypermethylation associated with Klinefelter syndrome and DNA hypomethylation associated with TS, which also shed light upon several candidate genes [114, 115].

## 3. Future Directions

X-linked diseases affect female patients differently resulting in a wide range of phenotype depending on their X-chromosome inactivation pattern. As seen in the review above, most of the studies mentioned are case studies and larger sample size could benefit exploration of the relationship between skewed X inactivation and phenotype severity. It is also important to note the cell type used when analyzing XCI skewing pattern, since it varies between different cell types even in the same individual. Also, it would be the best to analyze samples from affected organs to explore the relationship between XCI skewing and phenotype severity in female heterozygotes. Targeted reactivation of normal allele on the Xi could be further explored to support the development of more treatment options, as shown in studies in rodent models and cell lines [116, 117].
